# Integrative Approach to Identifying System-Level Mechanisms of Chung-Sang-Bo-Ha-Hwan’s Influence on Respiratory Tract Diseases: A Network Pharmacological Analysis with Experimental Validation

**DOI:** 10.3390/plants12173024

**Published:** 2023-08-22

**Authors:** Sa-Yoon Park, Kang-Sub Kim, Won-Yung Lee, Chang-Eop Kim, Sullim Lee

**Affiliations:** 1 Department of Physiology, College of Korean Medicine, Gachon University, Seongnam 13120, Republic of Korea; psy9228@gmail.com (S.-Y.P.); lwy21@gachon.ac.kr (W.-Y.L.); 2 College of Korean Medicine, Gachon University, Seongnam 13120, Republic of Korea; kangsub@gachon.ac.kr; 3 Department of Life Science, College of Bio-Nano Technology, Gachon University, Seongnam 13120, Republic of Korea

**Keywords:** Chung-Sang-Bo-Ha-Hwan, network pharmacological analysis, respiratory tract disease

## Abstract

Chung-Sang-Bo-Ha-Hwan (CSBHH) is an herbal prescription widely used to treat various chronic respiratory diseases. To investigate the system-level treatment mechanisms of CSBHH in respiratory tract diseases, we identified 56 active ingredients of CSBHH and evaluated the degree of overlap between their targets and respiratory tract disease-associated proteins. We then investigated the respiratory tract disease-related signaling pathways associated with CSBHH targets. Enrichment analysis showed that the CSBHH targets were significantly associated with various signaling pathways related to inflammation, alveolar structure, and tissue fibrosis. Experimental validation was conducted using phorbol-12-myristate-13-acetate (PMA)-stimulated NCI-H292 cells by analyzing the mRNA expression levels of biomarkers (IL-1β and TNF-α for inflammation; GSTP1, GSTM1, and PTEN for apoptosis) derived from network pharmacological analysis, in addition to the mucin genes MUC5AC and MUC2, to investigate the phlegm-expelling effect of CSBHH. The mRNA expression levels of these genes were consistent with network pharmacological predictions in a concentration-dependent manner. These results suggest that the therapeutic mechanisms of CSBHH in respiratory tract diseases could be attributed to the simultaneous action of multiple active ingredients in the herbal prescription.

## 1. Introduction

Respiratory tract diseases represent a broad spectrum of illnesses and afflictions that compromise the body’s primary gas exchange system. These diseases impact the upper or lower respiratory tract and encompass conditions ranging from common ailments, such as influenza and rhinitis, to severe diseases, such as pneumonia and chronic obstructive pulmonary disease (COPD). The global burden of respiratory tract diseases is substantial and escalating, contributing to an array of socioeconomic and public health challenges due to emergent threats, such as novel viruses such as severe acute respiratory syndrome coronavirus 2 (SARS-CoV-2), the causative agent of the coronavirus disease (COVID-19) pandemic, and escalating environmental pollutants, such as fine particulate matter [1]. These escalating threats pose a looming potential for more incidences of respiratory tract diseases in the future and have already begun to transform the socioeconomic and public health landscape worldwide.

Herbal medicines are widely used in Asian countries and are well received by patients. Chung-Sang-Bo-Ha-Hwan (CSBHH) or Chung-Sang-Bo-Ha-Tang (CSBHT) has been used for centuries to treat chronic lung diseases, such as asthma, in Korea [2,3]. Both CSBHH and CSBHT consist of 18 identical medicinal herbs, with the only distinction lying in their respective formulations. Previous clinical findings indicated that CSBHT improved Quality-of-Life Questionnaire for Adult Korean Asthmatics scores, increased forced expiratory volumes 1.0 and peak expiratory flow rates, and reduced the need for steroids and h2 agonists [4]. In a previous study, a modified herbal formula derived from CSBHT, PM014, was effective in a murine COPD model [5], cockroach allergen-induced allergic airway inflammation [6], and bleomycin-induced pulmonary fibrosis [7]. Despite its historical use and observed benefits, however, the therapeutic mechanisms of CSBHH have been largely unknown.

Network pharmacology, which is derived from systems biology, is a powerful tool for identifying rational drug targets and repurposing drugs [8]. This approach is beneficial for elucidating the mechanisms of disease treatment within interconnected biological networks, thereby overcoming the limitations of the single-target paradigm [9]. Network pharmacology is particularly useful for investigating the complex mechanisms of herbal medicines that frequently possess multi-compound and targeting properties. Network based approaches can also be used to investigate novel drug indications and their underlying action mechanisms. Specifically, disease treatment mechanisms can be accurately identified by considering the biological functions through which target proteins modulate the functions of disease-related proteins [10]. Therefore, this study demonstrated that drug–disease associations and their pivotal mechanisms can be precisely identified by considering the effects of drugs and diseases at the multiscale network level. These findings suggest that network pharmacology can provide novel opportunities for discovering the key mechanisms and active compounds in herbal medicines for treating respiratory tract diseases.

In the realm of traditional medicine, CSBHH has been employed for centuries to treat various respiratory diseases. Despite its historical use and observed benefits, the therapeutic mechanisms of CSBHH remain largely unclear. This study pioneered the application of a multiscale network-level approach with experimental validation to unravel the therapeutic effects and mechanisms of action of CSBHH in respiratory tract diseases. We collected information on the detectable components of CSBHH and their experimentally validated targets. By analyzing the collected dataset, we identified vital targets directly related to respiratory tract diseases that were significantly associated with disease-related signaling pathways. Furthermore, we investigated the effects of CSBHH on the targets related to respiratory diseases using phorbol-12-myristate-13-acetate (PMA)-stimulated human respiratory epithelial cells and comprehensively validated the protective effects of CSBHH in vitro. By doing so, we aimed to bridge the gap between traditional knowledge and modern scientific understanding, thereby providing a comprehensive strategy for elucidating the effects of CSBHH on various respiratory tract diseases and their system-level mechanisms.

## 2. Results

### 2.1. Identification of CSBHH Compounds and Targets

**Figure 1:**
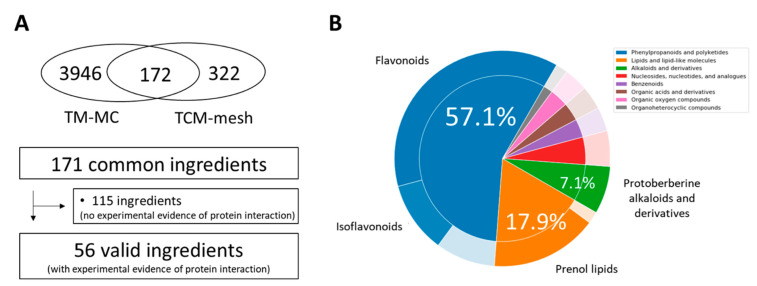
The selection process for Chung-Sang-Bo-Ha-Hwan (CSBHH)-active ingredients and their chemical distribution. (**A**) The flowchart of selecting the active ingredients of CSBHH. (**B**) Distribution of CSBHH-active ingredients across different chemical super-classes and classes obtained from ClassyFire. The inner and outer circles represent the proportions of superclass and classes, respectively.

We identified the active ingredients of 18 herbs constituting CSBHH, which have been recorded and detected experimentally [11]. We identified 56 active ingredients from the 12 herbs constituting CSBHH (Figure 1A); six herbs were excluded in the following analyses according to the criteria described in Section 4. The herbs shared no common active ingredients, indicating that each herb contained different active ingredients. To investigate the chemical diversity, we identified CSBHH-active ingredient super-classes and classes using Classyfire [12]. The results showed that the CSBHH-active ingredients were distributed across 8 super-classes and 16 classes. Among the super-classes, ‘phenylpropanoids and polyketides’, ‘lipids and lipid-like molecules’, and ‘alkaloids and derivatives’ were the top three super-classes, with 32, 10, and 4 active ingredients, respectively. Among the 16 classes, flavonoids, prenol lipids, and isoflavonoids were the top three classes, with 21, 9, and 6 active ingredients, respectively (Figure 1B).

We identified experimentally validated protein targets of CSBHH-active ingredients from the therapeutic target database (TTD), STITCH, DrugBank, and a dataset assembled by Huang et al. [13]. We assembled 3414 compound–protein interactions (CPIs) between the 56 active ingredients and 1081 protein targets. Among the target proteins, caspase 3 (CASP3), bcl-2-associated X (BAX), tumor necrosis factor (TNF), mitogen-activated protein kinase (MAPK)14, and V-rel avian reticuloendotheliosis viral oncogene homolog A (RELA) showed the strongest interaction with the CSBHH-active ingredients (45, 35, 34, 33, and 30 CPIs, respectively).

### 2.2. Degree of Overlap between CSBHH Targets and Respiratory Tract Disease-Related Proteins

To investigate the association between CSBHH and respiratory tract diseases, we evaluated the degree of overlap between the CSBHH targets and respiratory tract disease-related proteins. The result showed that approximately one-third of proteins (34/107) were associated with CSBHH targets. We conducted a hypergeometric test to check whether the observed number of overlapping targets was higher than the random expectations. The values of random expectation were obtained by randomly selecting targets equal to the number of targets from the assembled CPI dataset and repeatedly calculating the number of overlapping targets between respiratory tract disease-related proteins and selected targets. We found that the observed value between CSBHH targets and respiratory tract disease-related proteins was significantly higher (2.80-fold) than the random expectations (*p* < 10^−10^).

The same analysis was performed for the targets of each herb and ingredient constituting the CSBHH. At the targets for each herb, the targets of most herbs were significantly associated with respiratory tract disease-related proteins (Table 1). Particularly, we found that the odds ratios for *Platycodonis Radix*, *Dioscoreae Rhizoma,* and *Poria Sclerotium* were 20.73, 13.82, and 8.98, respectively. Moreover, the targets of *Glycyrrhizae Radix et Rhizoma*-, *Scutellariae Radix*-, *Coptidis Rhizoma*-, *Moutan Radicis Cortex*-, *Dioscoreae Rhizoma*-, and *Platycodonis Radix*-active ingredients were closely related to the respiratory tract disease-related proteins (Table 2). These results revealed that the herbs or active ingredients could play a key role in treating respiratory tract diseases with CSBHH.

### 2.3. Enriched Pathways and Biological Processes of CSBHH

**Figure 2:**
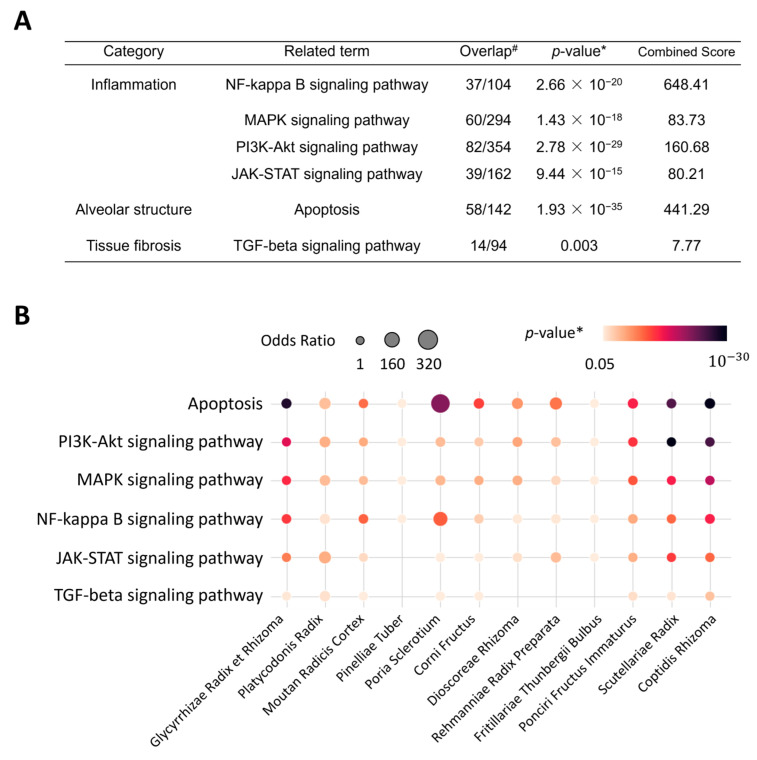
Enriched signaling pathways for Chung-Sang-Bo-Ha-Hwan (CSBHH) and its herbs. (**A**) Enriched signaling pathways related to CSBHH targets. (**B**) Associations between CSBHH herb targets and signaling pathways. # Number of overlapped proteins/targets, * *p*-values are adjusted by Bonferroni corrections.

We investigated whether and which respiratory tract disease-related signaling pathways were associated with CSBHH targets. The results of the enrichment analysis showed that CSBHH targets were significantly associated with various respiratory tract disease-related signaling pathways involved in inflammation, the alveolar structure, and tissue fibrosis (Figure 2A). To identify the herbs primarily involved in these mechanisms, we iteratively conducted the same analysis for all herb targets and related pathways (Figure 2B). The results showed that the targets of all herbs were significantly related to apoptosis and the phosphoinositide 3-kinase (PI3K)-Akt, MAPK, and nuclear factor (NF)-κB signaling pathways. Specifically, we found a pair of herb–target signaling pathways where the targets overlapped more than 50 times compared to the chance level, such as *the Poria Sclerotium*–apoptosis (odds ratio: 361.89), *Poria Sclerotium*–NF-κB signaling pathway (138,03), *Rehmanniae Radix Preparata*–apoptosis (84.62), and *Platycodonis Radix*–JAK-STAT signaling pathway (78.94) pairs. These results suggest that CSBHH herbs are beneficial against respiratory tract diseases by comprehensively regulating the related signaling pathways.

### 2.4. Network Analysis of the Mechanisms of CSBHH against Respiratory Tract Diseases

**Figure 3:**
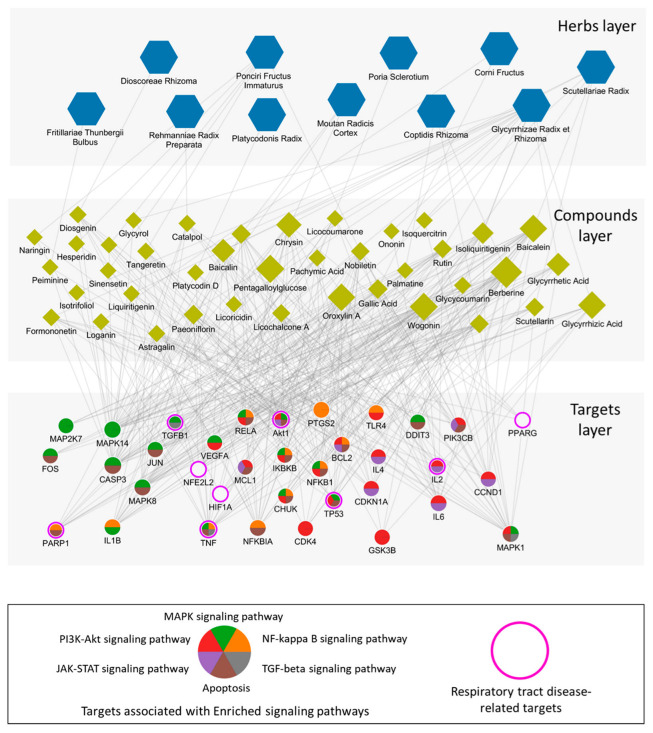
Representative compound–target network for Chung-Sang-Bo-Ha-Hwan (CSBHH). Hexagons, diamonds, and circles denote the herbs, compounds, and protein targets, respectively. The target proteins related to respiratory tract disease-related functional modules and respiratory tract diseases are presented by the body color and border, respectively.

A biological molecular network targeted by CSBHH was constructed to uncover the relationships among herbs, active ingredients, protein targets, and respiratory tract diseases. To elucidate the potential key mechanisms, we constructed the herb–active ingredient–target subnetwork focusing on targets that have a degree of overlap with CSBHH-active ingredients of at least seven (top 10% of the target degrees of overlap) (Figure 3). The network consisted of 11 herbs, 43 active ingredients, and 46 target proteins, with 486 interactions between them (43 herb–active ingredient associations and 443 active ingredient–target associations). We found that most CSBHH-active ingredients interacted with targets such as CASP3, TNF, MAPK14, RELA, Akt1, MAPK1, MAPK8, BCL2-associated agonist of cell death (BAD), NFKB1, interleukin 6 (IL6), prostaglandin-endoperoxide synthase 2 (PTGS2), NFKB Inhibitor Alpha (NFKBIA), CASP9, and (IL-1β). Among the CSBHH targets, Akt1, transforming growth factor beta (TGFB1), TNF, IL2, tumor protein p53 (TP53), and poly (ADP-ribose) polymerase 1 (PARP1) were respiratory tract disease-related targets (direct relationship) and were involved in enriched signaling pathways (indirect relationship). These results suggest that the therapeutic mechanisms of CSBHH in respiratory tract diseases could be attributed to the simultaneous action of multiple active ingredients in the herbal formula.

### 2.5. Effects of CSBHH on NCI-H292 Cell Viability

**Figure 4:**
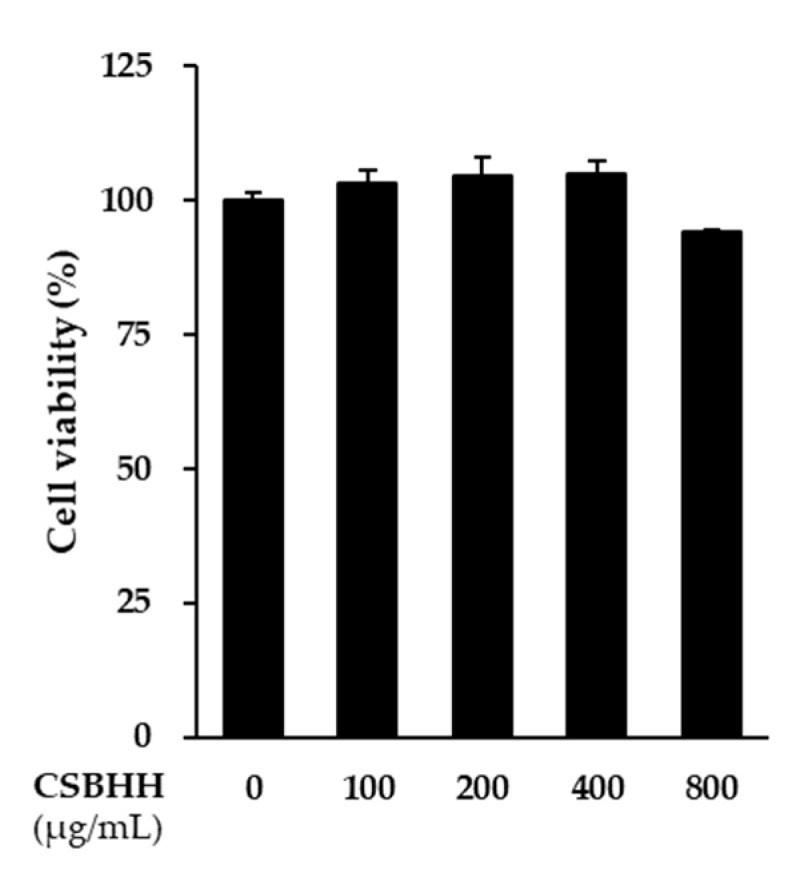
Effect of Chung-Sang-Bo-Ha-Hwan (CSBHH) on NCI-H292 cell viability. The cells (2 × 10^4^ cells/well) were cultured in a 96-well plate for 24 h. Thereafter, the cells were treated with CSBHH at a specific concentration for 24 h. Cell viability was determined using an Ez-Cytox kit. Data are presented as the mean ± SEM (*n* = 3).

We investigated the cytotoxicity on NCI-H292 cells to determine the appropriate CSBHH concentration range for additional experiments. Since CSBHH was not toxic at concentrations below 800 μg/mL, 200, 400, and 800 μg/mL CSBHH were used (Figure 4).

### 2.6. Effect of CSBHH on PMA-Induced mRNA Expression in NCI-H292 Cells

To investigate the inhibitory effect of CSBHH on PMA-induced mRNA expression, the cells were pre-treated with 200, 400, or 800 μg/mL CSBHH and then treated with 100 nM PMA for 3 or 24 h. After 3 h of PMA treatment, IL-1β, TNF-α, mucin (MUC)5AC, and MUC2 mRNA expression levels were measured, and 24 h after treatment, glutathione S-transferase pi (GSTP1), glutathione S-transferase mu 1 (GSTM1), and phosphatase and tensin homolog (PTEN) mRNA expression levels were measured.

**Figure 5:**
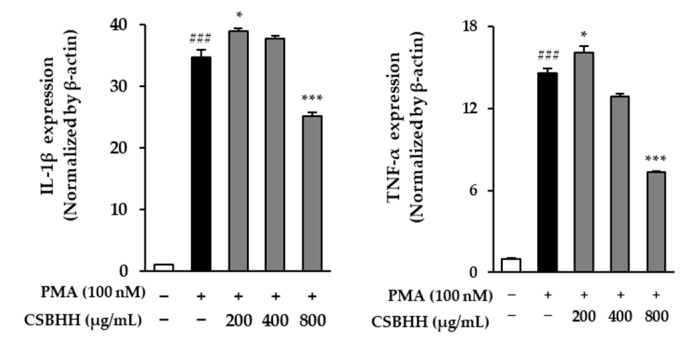
Effect of Chung-Sang-Bo-Ha-Hwan (CSBHH) on phorbol 12-myristate 13-acetate (PMA)-induced IL-1β and TNF-α mRNA expression in NCI-H292 cells. The mRNA expression was assessed by qRT-PCR analysis. Data are presented as the mean ± SEM (*n* = 2). ^###^ *p* < 0.001 compared to the untreated cells; * *p* < 0.05 and *** *p* < 0.001 compared to the PMA-treated cells.

IL-1β and TNF-α mRNA expression in the PMA-stimulated cells increased by 34.75-fold and 14.60-fold (*p* < 0.001), respectively, compared to that in the untreated cells. However, they were reduced by 0.27-fold and 0.50-fold (*p* < 0.001), respectively, in the 800 μg/mL CSBHH-treated cells compared to those in the PMA-stimulated cells (Figure 5).

**Figure 6:**
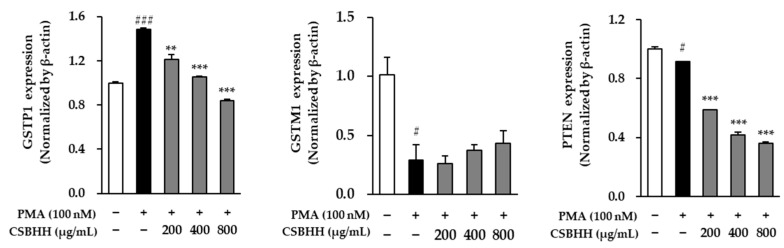
Effect of Chung-Sang-Bo-Ha-Hwan (CSBHH) on phorbol 12-myristate 13-acetate (PMA)-induced GSTP1, GSTM1, and PTEN mRNA expression in NCI-H292 cells. The mRNA expression was assessed by qRT-PCR analysis. Data are presented as the mean ± SEM (*n* = 2). ^#^ *p* < 0.05 and ^###^ *p* < 0.001 compared with the untreated cells; ** *p* < 0.01 and *** *p* < 0.001 compared with the PMA-treated cells.

The GSTP1 mRNA expression in the PMA-stimulated cells increased by 34.75-fold (*p* < 0.001) compared to that in the untreated cells. However, it was reduced by 0.43-fold (*p* < 0.001) in the 800 μg/mL CSBHH-treated cells compared to that in the PMA-stimulated cells. Interestingly, the mRNA expression of GSTM1 and PTEN in the PMA-stimulated cells decreased by 0.29-fold (*p* < 0.05) and 0.92-fold (*p* < 0.05), respectively, compared to that in the untreated cells. Additionally, the GSTM1 mRNA expression increased by 1.48-fold in the cells treated with 800 μg/mL CSBHH compared to that in the PMA-stimulated cells, and PTEN mRNA expression decreased by 0.61-fold (*p* < 0.001) (Figure 6).

**Figure 7:**
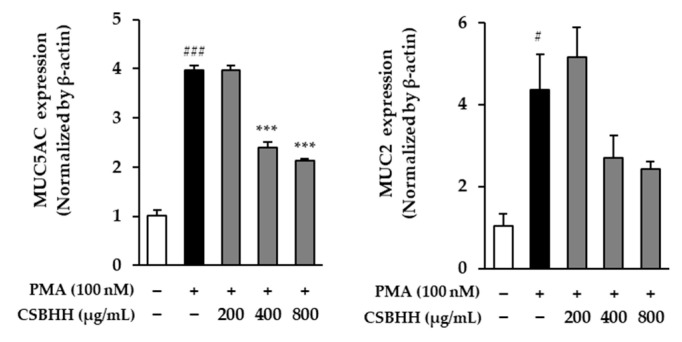
Effect of Chung-Sang-Bo-Ha-Hwan (CSBHH) on phorbol 12-myristate 13-acetate (PMA)-induced MUC5AC and MUC2 mRNA expression in NCI-H292 cells. The mRNA expression was assessed by qRT-PCR analysis. Data are presented as the mean ± SEM (*n* = 2). ^#^ *p* < 0.05 and ^###^ *p* < 0.001 compared to the untreated cells; *** *p* < 0.001 compared to the PMA-treated cells.

MUC5AC and MUC2 mRNA expression increased by 3.97-fold (*p* < 0.001) and 4.36-fold (*p* < 0.05), respectively, in PMA-stimulated cells compared to that in untreated cells. However, they decreased by 0.47-fold (*p* < 0.001) and 0.44-fold, respectively, in the 800 μg/mL CSBHH-treated cells compared to those in the PMA-stimulated cells (Figure 7).

## 3. Discussion

In this study, we successfully demonstrated the therapeutic effects of CSBHH against respiratory tract diseases by combining network pharmacological analyses and experimental validation. We identified the herbs and active ingredients that play key roles in treating respiratory tract diseases by considering the degree of overlap between CSBHH targets and respiratory tract disease-related proteins. We revealed the underlying mechanisms of CSBHH in respiratory tract diseases by identifying enriched respiratory tract disease-related signaling pathways. Particularly, by focusing on targets that have a degree of overlap with CSBHH-active ingredients equal to or greater than seven in the herb–active ingredient–target network, we identified key protein targets both directly and indirectly related to diseases. These findings were systematically validated in vitro. Our study provides a solid foundation for accelerating the potential use of CSBHH as a predictor of various respiratory tract diseases.

Network pharmacological analysis revealed complex relationships among the compounds, targets, and biological effects of CSBHH on respiratory tract diseases. This comprehensive analysis provides insights into the potential interactions between CSBHH-active ingredients and their protein targets in respiratory tract diseases. By constructing a compound–target network for CSBHH using various databases, we initially identified 3414 CPIs involving 56 CSBHH-active ingredients and 1081 protein targets. Among the 107 respiratory tract disease-associated proteins, approximately one-third (34/107) were associated with CSBHH targets, indicating the statistical significance of CSBHH, its individual herbs, and key active ingredients in respiratory tract diseases. Our subsequent analysis revealed that individual herb targets showed significant overlap with respiratory tract disease-related targets, such as *Platycodonis Radix*, *Dioscoreae Rhizoma,* and *Poria Sclerotium* (20.73-, 13.82-, and 8.98-fold overlap, respectively).

*Platycodonis Radix* possesses anti-inflammatory, anti-cancer, anti-diabetic, and anti-obesity properties [14]. Platycodin D, a physiologically active *Platycodonis Radix* component, attenuates airway inflammation through the NF-κΒ signaling pathway in an ovalbumin (OVA)-induced asthmatic mouse model. Platycodin D inhibits eosinophilic inflammation, mucus production, and the production of T helper 2 (Th2) cytokines, such as IL-4, IL-5, and IL-13, in the bronchial mucosa of an OVA-induced asthmatic mouse model [15,16,17].

*Dioscoreae Rhizoma* has anti-diabetic, antioxidant, and anti-inflammatory effects [18,19,20]. It also reduces IL-1β, TNF-α, cyclooxygenase 2 (COX-2), and inducible nitric oxide synthase (iNOS) expression in human fibroblast-like synovial cells [19]. Diosgenin, a physiologically active *Dioscoreae Rhizoma* component, reduces the expression of inflammatory factors, such as IL-1β, TNF-α, and IL-6, in the OVA-induced asthmatic mouse model and primary tracheal epithelial cells and downregulates the NF-κΒ signaling pathway by activating glucocorticoid receptors [21,22].

*Poria Sclerotium*, used as an herbal medicine, has various activities such as anti-inflammatory, anti-bacterial, anti-tumor, and immune-enhancing properties [23,24,25]. Polysaccharides isolated from *Poria Sclerotium* induced nitric oxide (NO) production and iNOS transcription through NF-κΒ/Rel activation in RAW264.7 cells. Moreover, regulating the type 1 T-helper (Th1)/type 2 T-helper (Th2) response in OVA-induced mice has been observed to aid in improving immunodeficiency and preventing allergic diseases, including allergic asthma [26,27,28].

*Glycyrrhizae Radix* has anticancer, antiviral, and anti-inflammatory effects [29,30,31]. 18β-Glycyrrhetinic acid (18β-GA), a physiologically active component of *Glycyrrhizae Radix*, inhibits allergic airway inflammation through the NF-κB signaling pathway [32]. Rutin downregulated the NF-κB and iNOS signaling pathways involved in the inflammatory response in cigarette smoke and ovalbumin (OVA)-induced asthmatic mouse models. It has been reported that formononetin reduces the expression of IL-1β, NF-κB, and MUC5AC in IL-13-stimulated JME/CF15 cells, thereby reducing inflammation and mucus formation [33,34]. It has also been reported that formononetin and licochalocone A attenuated airway inflammation and oxidative stress in an OVA-induced asthmatic mouse model [35,36,37].

*Scutellariae Radix* has antioxidant, anti-inflammatory, and anti-obesity effects [38]. Oroxylin, a physiologically active component of *Scutellariae Radix*, downregulates the NF-κB signaling pathway in LPS-induced macrophages [39]. It has also been reported to reduce the expression levels of IL-1b and TNF-α in the lung tissue of a COPD mouse model and suppress airway inflammation and mucus production by inhibiting NF-κB activity in an OVA-induced asthmatic murine model [40,41,42]. Chrysin has been reported to inhibit the production of MUC5AC in NCI-H292 cells induced by PMA, EGF, and TNF-α [43,44,45]. Wogonin reduces the production of MUC5AC and inhibits the NF-kB and MAPK signaling pathways in TNF-α- and EGF-induced NCI-H292 cells. It has also been reported to alleviate airway inflammation by inhibiting IL-4/STAT6 signaling in an OVA-induced asthmatic mouse model [46,47,48].

*Coptidis rhizoma* has anticancer, anti-inflammatory, and expectorant effects [49,50]. Berberine, a physiologically active component of *Coptidis rhizoma*, downregulates the MAPK signaling pathway in breast cancer cells, induces apoptosis, and inhibits cancer cell proliferation. It suppresses the growth and invasion of breast cancer cells by downregulating the EGFR/MEK/ERK pathway [51,52,53]. It has also been reported to reduce TNF-α and IL-1β in the bronchoalveolar lavage fluid (BALF) of mice induced by cigarette smoke and to suppress the production of MUC5AC in human airway epithelial cells through MAPKs ERK and p38 [54,55,56].

*Moutan Radicis Cortex* has anticancer, antioxidant, and anti-inflammatory effects [57,58]. Paeonol, a physiologically active component of *Moutan Radicis Cortex*, has been reported to have anti-inflammatory and anticoagulant effects in an LPS-induced acute lung injury rat model and to reduce IL-1β levels in the BALF of cigarette smoke-induced mice [59,60].

Through an over-representation analysis based on the disease-related signaling pathways, we discovered that inflammation and the alveolar structure were the main categories, since all herbs have a significant relationship with PI3K–Akt, MAPK, NF-κΒ signaling, and apoptosis pathways. Furthermore, by focusing on 46 targets that interacted with seven or more active ingredients, we found that the key targets were both respiratory tract disease-related targets (direct relationship) and involved in enriched signaling pathways (indirect relationship), such as Akt1, TGFB1, TNF, IL2, TP53, and PARP1.

The potential targets of CSBHH related to the PI3K–Akt, MAPK, NF-κΒ signaling, and apoptosis pathways were summarized using KEGG Mapper (Supplementary Figure S1). In the context of the PI3K-Akt signaling pathway, CSBHH exhibits a multi-targeted approach that interacts with several key targets within this pathway. Notably, pink-colored targets, which are activated by CSBHH, play crucial roles in various cellular processes. For example, *Akt1*, a serine/threonine-specific protein kinase, is involved in processes such as glucose metabolism, apoptosis, cell proliferation, transcription, and cell migration. Furthermore, CSBHH interacted with *PTEN*, a major negative regulator of the PI3K-Akt pathway. This interaction could potentially modulate PI3K-Akt signaling, thereby influencing cell survival, growth, and proliferation. In the MAPK signaling pathway, CSBHH appears to specifically interact with *MAPK14* (*p38-α*), *MAPK1* (*ERK2*), and *MAPK8* (*JNK1*). These interactions suggest a potential modulation of cellular processes, such as the stress response, inflammation, cell proliferation, differentiation, and apoptosis, which could contribute to the treatment of respiratory diseases. Additionally, CSBHH’s interaction with *TNF* and *CASP3* could influence the inflammatory response and apoptosis, respectively. Within the NF-kappa B signaling pathway, CSBHH demonstrates interactions with key targets—specifically, *RELA* and *NFKB1*. The modulation of cellular processes, such as immunity, inflammation, and cell survival, by CSBHH underscores its therapeutic potential. In the apoptosis pathway, CSBHH has been found to interact with several key targets, including *CASP3*, *TNF*, *MAPK14*, *RELA*, *Akt1*, *MAPK1*, *MAPK8*, *BAD*, *NFKB1*, *IL6*, *PTGS2*, *NFKBIA*, and *CASP9*. These interactions indicate that CSBHH may have the ability to modulate the apoptosis process, which could potentially be beneficial in the treatment of respiratory diseases. Altogether, these findings underscore the potential of CSBHH in treating respiratory diseases through a multi-targeted approach, influencing various signaling pathways and cellular processes.

These findings have guided the in vitro experimental trials while highlighting the complex and multifaceted nature of the potential for treating respiratory tract diseases. Considering the results of the in silico network pharmacological analysis, we investigated several inflammation- and apoptosis-related genes for experimental validation. We investigated the inhibitory effects of CSBHH on PMA-induced mRNA expression and found that *IL-1β*, *TNF-α*, *GSTP1*, *MUC5AC*, and *MUC2* gene expression increased in PMA-stimulated NCI-H292 cells. Our results showed that the PMA-induced increased *IL-1β*, *TNF-α*, *GSTP1*, *MUC5AC*, and *MUC2* mRNA expression tended to decrease with CSBHH treatment. However, *GSTM1* expression was decreased and increased in the PMA- and CSBHH-treated groups, respectively.

In this study, IL-1 and TNF-α levels were evaluated as indicators of inflammation. The IL-1 family includes key cytokines involved in fever, inflammation, and the innate immune response [61]. IL-1β is produced as a precursor protein and has pro-inflammatory activity that induces the production of pro-inflammatory mediators [62,63]. TNF-α induces various cellular responses including cell survival, differentiation, and proliferation, regulates inflammatory responses, and is also involved in inflammatory and autoimmune diseases [64,65]. In NCI-H292 cells, apoptosis and antioxidant properties are regulated through the ROS/MAPK signaling pathway [66]. Therefore, CSBHH should also be effective in inflammatory and immune responses by reducing the expression of the pro-inflammatory cytokines IL-1β and TNF-α (Figure 5).

GSTP1, GSTM1, and PTEN play key roles in alveolar apoptosis [67,68,69]. GSTP1, a glutathione S-transferase subtype, maintains the cellular oxidation balance and regulates cell proliferation and apoptosis, particularly through JNK interaction [70,71,72,73]. Similarly, GSTM1, another glutathione S-transferase subtype, protects cells from oxidative stress, detoxifies harmful substances, and inhibits apoptosis by interacting with apoptosis signal-regulating kinase 1 (ASK1) [74]. PTEN antagonizes the PI3K–protein kinase B (AKT)–mammalian target of rapamycin (mTOR) pathway, thereby regulating cell survival and proliferation [75,76]. GSTP1-mediated apoptosis regulation and the role of GSTM1 in oxidative stress and detoxification have been confirmed by studies showing that GSTP1 inhibition causes JNK activation and cisplatin-induced apoptosis [67,70]. Additionally, GSTM1 binds to ASK1 and inhibits ASK1-dependent apoptosis [77,78]. In NCI-H292 cells, apoptosis is regulated through the ERK 1/2 signaling pathway [79]. With decreased GSTP1 and increased GSTM1 expression in CSBHH-treated groups, apoptosis induction was anticipated (Figure 6).

PTEN antagonizes the PI3K–AKT–mTOR pathway and regulates numerous cellular processes including cell survival and proliferation. Therefore, PTEN regulation is an important factor in cancer development because the impairment of PTEN function triggers mechanisms such as cell survival and proliferation [75,76]. Similarly, PTEN expression decreased and increased in NCI-H292 cells stimulated with cigarette smoke and treated with rosiglitazone, a peroxisome proliferator-activated receptor-γ (PPAR-γ) agonist, respectively [80]. PTEN expression in PMA-induced NCI-H292 cells was similar to that in the untreated cells and increased in the 15-hydroxyeicosatetraenoic acid-treated cells, which is effective in chronic airway inflammation and pharmacological treatment [81]. In MCF-7, another cancer cell line, PTEN expression was decreased by bisphenol A or 17β-estradiol and increased by curcumin [82]. Moreover, the combined treatment with quercetin or luteolin along with 5-fluorouracil, which is used as an anti-cancer drug, increased PTEN expression in the colon cancer cell line HT-29 and improved its anti-cancer effect [83]. However, unlike previous experimental results, the PTEN levels in our study showed a decreasing trend. Although these previous studies investigated the effect of a single compound, the sample we used, CSBHH, is an herbal medicine prescription composed of various herbs; therefore, it is thought that a complex reaction of each component occurred, and thus, the result was obtained (Figure 6).

In addition, we experimentally evaluated the mucin genes *MUC5AC* and *MUC2* as well as five genes derived through network pharmacological analysis. Clinically, CSBHH has been widely used to treat various chronic respiratory diseases, particularly those related to phlegm, with the effect of dispelling phlegm and relieving panting [84]. The hypersecretion of airway mucus is an important physiological and clinical symptom of various respiratory tract diseases, such as chronic bronchitis and asthma, and airway stimuli, such as antigens, bacteria, and particles, upregulate the *MUC5AC* and *MUC2* genes [85]. Therefore, reducing *MUC5AC* and *MUC2* expression may effectively alleviate excessive mucus production in respiratory diseases. Mucus is composed of mucin proteins, and MUC5AC is the major mucin protein secreted from airway epithelial cells, such as NCI-H292 cells. Therefore, we included the mucin genes, *MUC5AC* and *MUC2*, among the 21 mucin genes discovered to date [86,87].

Numerous studies have reported the efficacy of CSBHT in expelling phlegm; CSBHT differs from CSBHH only in its formulation. In an OVA-induced allergic asthma model, CSBHT increased mucin sequestration from the bronchi and improved eosinophil infiltration into the tracheal mucosa [88,89]. In addition, the increased goblet cells in OVA-induced mice decreased, similar to dexamethasone treatment, which prevents mucus production in the airway [2]. Similar to anti-inflammation and apoptosis, MUC5AC and MUC2 expression is regulated through the MAPK and NF-κB signaling pathways [90,91,92]. Our results showed that CSBHH decreased MUC5AC and MUC2 expression in PMA-induced NCI-H292 cells, which agreed with the results of previous studies (Figure 7). Therefore, reducing MUC5AC and MUC2 expression may effectively alleviate excessive mucus production in respiratory tract diseases.

Medications used to treat respiratory diseases such as asthma and COPD include controllers and relievers. Controller medications are used for routine maintenance and treatment to control the symptoms and reduce airway inflammation. Reliever medications are used as needed to relieve breakthrough symptoms or the exacerbation of symptoms [93]. Combinations of inhaled corticosteroids (ICS) and long-acting β-agonists (LABA), leukotriene receptor antagonists, and short-acting β-agonists (SABA), etc. are used, mainly bronchodilators and glucocorticoids, including β2 receptor agonists and anticholinergic drugs. β2 receptor agonists have side effects such as a rapid heart rate, metabolic disturbances, and muscle tremors, while anticholinergic drugs have side effects such as cognitive problems and an impaired heart rhythm [94,95]. In addition, long-term systemic corticosteroid (SCS) therapy or high-dose inhaled corticosteroid use induces osteoporosis, adrenal suppression, and psychiatric disorders [11,96,97].

In herbal medicine prescriptions, the active ingredients contained in each medicinal material are extracted through a decocting method for various medicinal materials. These active ingredients interact with each other to create a synergistic effect that is suitable for treating complex diseases through various activities and has few side effects. These active ingredients interact with each other to create a synergistic effect, which is suitable for treating complex diseases through various activities and has few side effects [98,99]. Kang et al. conducted in vitro, animal, and human studies of CSBHT in Korea and reported that CSBHT and its variants are being used effectively in clinical practice for allergic asthma and non-allergic asthma [11]. Thus, CSBHH has the advantage of being effective in improving symptoms such as cough and sputum as well as anti-inflammatory effects and lung function.

In light of our findings, we believe that CSBHH has significant potential as a novel treatment option for respiratory diseases. Our study sheds light on the therapeutic effects and mechanisms of action of CSBHH and contributes to a deeper understanding of its role in the treatment of respiratory diseases. Looking ahead, we envision a future in which traditional medicines such as CSBHH are integrated into mainstream healthcare, providing patients with more diverse and effective treatment options. We anticipate that our findings will spur further research into the therapeutic mechanisms of traditional medicines, potentially leading to the discovery of new treatment strategies for respiratory diseases. However, we acknowledge that our study was at the beginning. The full potential of CSBHH and other traditional medicines can only be realized through continued research and rigorous clinical trials. We hope that our work will serve as a stepping stone for future studies aimed at exploring the untapped potential of traditional medicines in the treatment of respiratory diseases.

## 4. Materials and Methods

### 4.1. Compound–Target Network Construction

A compound–target network is a bipartite network where nodes are defined as compounds or targets, and the edges between the compounds and targets are defined as interactions between them. A network was constructed by identifying active ingredients and their target proteins. We considered only the active ingredients of CSBHH herbs commonly recorded in TCM-mesh and TM-MC [100,101]. The TCM-mesh provides comprehensive herb–active ingredient information obtained from the TCM Database@Taiwan and TCMID with PubChem CID. TM-MC provides information on medicinal ingredients selected by Korean medicine doctors and biologists by reading chromatographic articles on medicinal plant materials. For our analysis, we considered ingredients that could be mapped to PubChem IDs and contained protein target information with experimental evidence.

The experimental targets of active CSBHH ingredients were obtained from data assembled by STITCH [102], DrugBank [103], TTD [104], and Huang et al. [13]. STITCH integrates the target information for 430,000 chemicals from disparate data sources. DrugBank is a comprehensive online database that contains information on drugs and drug targets. The TTD provides comprehensive information about known and explored targets, the targeted disease, pathway information, and the corresponding drugs directed at each target. Huang et al. assembled the direct and indirect compound–protein interactions of natural products from several databases.

### 4.2. Enrichment Analysis

The CSBHH target-associated pathways and biological processes were identified by enrichment analysis using Enrichr [105]. Enrichr computes enrichment by assessing multiple gene-set libraries (such as gene ontology (GO), KEGG, and Online Mendelian Inheritance in Man (OMIM)) and calculates adjusted *p*-values and combined scores for the target genes. The combined score was calculated using the logarithm of the product of the *p*-value and the z-score. Bonferroni correction was applied to rectify the family-wise errors generated in multiple tests.

### 4.3. Disease-Related Proteins and Pathways

The respiratory tract disease-related proteins and signaling pathways were retrieved to understand the mechanisms of action of CSBHH. The details of disease-related proteins were obtained from a comparative toxicogenomics database (CTD) [106]. We considered only manually curated associations by experts labeled as therapeutic, which refers to target proteins considered as treatment targets for the disease.

The disease-related signaling pathway and its type were identified in the Safety and Effectiveness Evaluation Guide of Health Functional Foods for enhancing the health of the respiratory tract published by the Ministry of Food and Drug Safety (Republic of Korea) as follows: the NF-kappa B signaling pathway (hsa04064), MAPK signaling pathway (hsa04010), PI3K–Akt signaling pathway (hsa04151), and Jak–STAT signaling pathway (hsa04630) for inflammation; the apoptosis pathway (hsa04210) for the alveolar structure; and the TGF-beta signaling pathway (hsa04350) for tissue fibrosis.

### 4.4. Cell Culture

Human mucoepidermoid pulmonary carcinoma cells, NCI-H292 (Korean Cell Line Bank, Seoul, Republic of Korea), were cultured in RPMI-1640 (Corning, Manassas, VA, USA) containing 10% fetal bovine serum (FBS; Atlas, Fort Collins, CO, USA) and 100 U/mL penicillin–streptomycin (Gibco, Grand Island, NY, USA) in 5% CO_2_ at 37 °C.

### 4.5. Sample Preparation

PMA (Sigma-Aldrich, St. Louis, MO, USA) was dissolved in 5 mM dimethyl sulfoxide stock solution (DMSO; Sigma-Aldrich), and the cells were stimulated for 3 or 24 h at a final concentration of 100 nM. CSBHH was dissolved in DMSO to a concentration of 200 mg/mL and diluted accordingly. For the cell experiments, 24 h-old NCI-H292 cells were starved overnight in serum-free RPMI-1640 medium and treated with PMA and CSBHH.

### 4.6. Cell Viability

NCI-H292 cells (2 × 10^4^ cells/well) were seeded in 96-well plates and allowed to adhere for 24 h. The cells were then treated with different concentrations of CSBHH for 24 h. Next, 100 μL of 10% EZ-Cytox solution (DoGenBio, Seoul, Republic of Korea) in serum-free RPMI-1640 was added to each well and incubated for 1 h. The OD450 nm was measured using a microplate reader (SPARK 10M; Tecan, Männedorf, Switzerland).

### 4.7. Total RNA Isolation and Quantitative Real-Time Polymerase Chain Reaction (qRT-PCR)

NCI-H292 cells (5 × 10^5^ cells/well) were seeded in six-well plates. The cells were then treated with a specific CSBHH concentration for 1 h after overnight starvation in a serum-free RPMI-1640 medium. The cells were then exposed to 100 nm PMA for 3 or 24 h. An RNeasy Mini Kit (Qiagen, Germantown, MD, USA) was used to isolate the total cellular RNA. The RevertAid First Strand cDNA Synthesis Kit (Thermo Fisher Scientific, Eugene, OR 97402, USA) was used to reverse-transcribe the RNA. PCR was performed using AccuPower^®^ 2X GreenStar™ qPCR Master Mix (Bioneer, Daejeon, Republic of Korea) on a Quant Studio 3 real-time PCR system (Applied Biosystems, Foster City, CA, USA) with forward and reverse primers (Table 3). β-Actin was used as the housekeeping gene. The amplification conditions were as follows: 50 °C for 2 min, 95 °C for 10 min, and 40 cycles of 95 °C for 15 s, 60 °C for 1 min, 95 °C for 15 s, 60 °C for 1 min, and 95 °C for 15 s.

## 5. Conclusions

Our study provides a system-level understanding of the therapeutic mechanisms of CSBHH, which is a widely used herbal prescription for chronic respiratory diseases. We identified 56 active compounds in CSBHH and found a substantial overlap between their targets and proteins associated with respiratory tract diseases. Notably, CSBHH targets were significantly linked to various signaling pathways associated with inflammation, the alveolar structure, and tissue fibrosis. Experimental validation using PMA-stimulated NCI-H292 cells showed that the mRNA expression levels of biomarkers (IL-1β, TNF-α, GSTP1, GSTM1, and PTEN) and mucin genes (MUC5AC and MUC2) were consistent with network pharmacological predictions, substantiating the effect of CSBHH on inflammation and apoptosis, as well as its phlegm-expelling effects. These results highlight a multi-target, multi-ingredient strategy underlying the therapeutic efficacy of CSBHH in respiratory tract diseases. Future studies should focus on elucidating the specific molecular interactions between the active components and their targets to further optimize the therapeutic potential of CSBHH.

## Figures and Tables

**Table 1:** Protein overlaps between Chung-Sang-Bo-Ha-Hwan (CSBHH) herb targets and respiratory tract disease-related proteins.

Herb Name	Number of Targets	Number of Overlapped Proteins	Odds Ratio ^#^	*p*-Value
*Glycyrrhizae Radix et Rhizoma*	235	14	5.35	4.4 × 10^−8^
*Platycodonis Radix*	13	3	20.73	9.6 × 10^−6^
*Moutan Radicis Cortex*	162	10	5.55	1.7 × 10^−6^
*Pinelliae Tuber*	264	3	1.02	0.34
*Poria Sclerotium*	20	2	8.98	1.3 × 10^−3^
*Corni Fructus*	65	5	6.91	8.0 × 10^−5^
*Dioscoreae Rhizoma*	26	4	13.82	8.5 × 10^−6^
*Rehmanniae Radix Preparata*	22	0	0.00	0.22
*Fritillariae Thunbergii Bulbus*	251	2	0.72	0.53
*Ponciri Fructus Immaturus*	90	4	3.99	3.2 × 10^−3^
*Scutellariae Radix*	302	16	4.76	3.3 × 10^−8^
*Coptidis Rhizoma*	186	12	5.80	1.3 × 10^−7^
Total (CSBHH)	1088	34	2.80	2.6 × 10^−9^

^#^ A ratio of the number of observed overlapping proteins compared with random selection.

**Table 2:** Protein overlaps between Chung-Sang-Bo-Ha-Hwan (CSBHH)-active ingredient targets and respiratory tract disease-related proteins.

Active Ingredient	Pubchem ID	Related Herb	Overlap ^#^	*p*-Value
Oroxylin	5320315	*Scutellariae Radix*	8/70	6.90 × 10^−8^
Berberine	2353	*Coptidis Rhizoma*	10/141	4.26 × 10^−7^
Paeonol	11092	*Moutan Radicis Cortex*	5/31	9.70 × 10^−7^
Chrysin	5281607	*Scutellariae Radix*	7/82	3.26 × 10^−6^
Diosgenin	99474	*Dioscoreae Rhizoma*	4/25	6.92 × 10^−6^
Platycodin D	162859	*Platycodonis Radix*	3/13	9.60 × 10^−6^
Rutin	5280805	*Glycyrrhizae Radix et Rhizoma*	5/46	1.08 × 10^−5^
Formononetin	5280378	*Glycyrrhizae Radix et Rhizoma*	4/29	1.49 × 10^−5^
Wogonin	5281703	*Scutellariae Radix*	7/108	2.53 × 10^−5^
Licochalcone A	5318998	*Glycyrrhizae Radix et Rhizoma*	4/39	6.62 × 10^−5^

^#^ Number of overlapping proteins/targets.

**Table 3:** Primer Sequences.

Gene	Forward Primer Sequence (5′–3′)	Reverse Primer Sequence (5′–3′)
IL-1β	CTGTCCTGCGTGTTGAAAGA	TTCTGCTTGAGAGGTGCTGA
TNF-α	TTCCCCAGGGACCTCTCTCTAATC	GAGGGTTTGCTACAACATGGGCTAC
GSTP1	GGCAACTGAAGCCTTTTGAG	GGCTAGGACCTCATGGATCA
GSTM1	CTGGGCATGATCTGCTACAATC	CAAAAGTGATCTTGTTTCCTGCAA
PTEN	TGGCTAAGTGAAGATGACAATCATG	TGCACATATCATTAC ACCAGTTCGT
MUC5AC	TCCACCATATACCGCCACAGA	TGGACGGACAGTCACTGTCAAC
MUC2	TGCCTGGCCCTGTCTTTG	CAGCTCCAGCATGAGTGC
β-actin	AGGAGAAGCTGTGCTACGTC	GGATGTCCACGTCACACTTC

Abbreviations: IL-1β, Interleukin 1 beta; TNF-α, Tumor necrosis factor alpha; GSTP1, Glutathione S-transferase pi 1; GSTM1, Glutathione S-transferase mu 1; PTEN, Phosphatase; tensin homolog MUC5AC, Mucin 5AC; and MUC2, Mucin 2.

## Data Availability

Not applicable.

## Supplementary Materials

The following supporting information can be downloaded at: https://www.mdpi.com/article/10.3390/plants12173024/s1.
