# Supplementary material for: Integrative Approach to Identifying System-Level Mechanisms of Chung-Sang-Bo-Ha-Hwan’s Influence on Respiratory Tract Diseases: A Network Pharmacological Analysis with Experimental Validation
Source: Plants (Basel). 2023 Aug 22;12(17):3024. doi: 10.3390/plants12173024 (PMC10489874; doi:10.3390/plants12173024)
Supplement: Supplementary file 1 [file plants-12-03024-s001.zip › plants-2529412-supplementary.pdf]

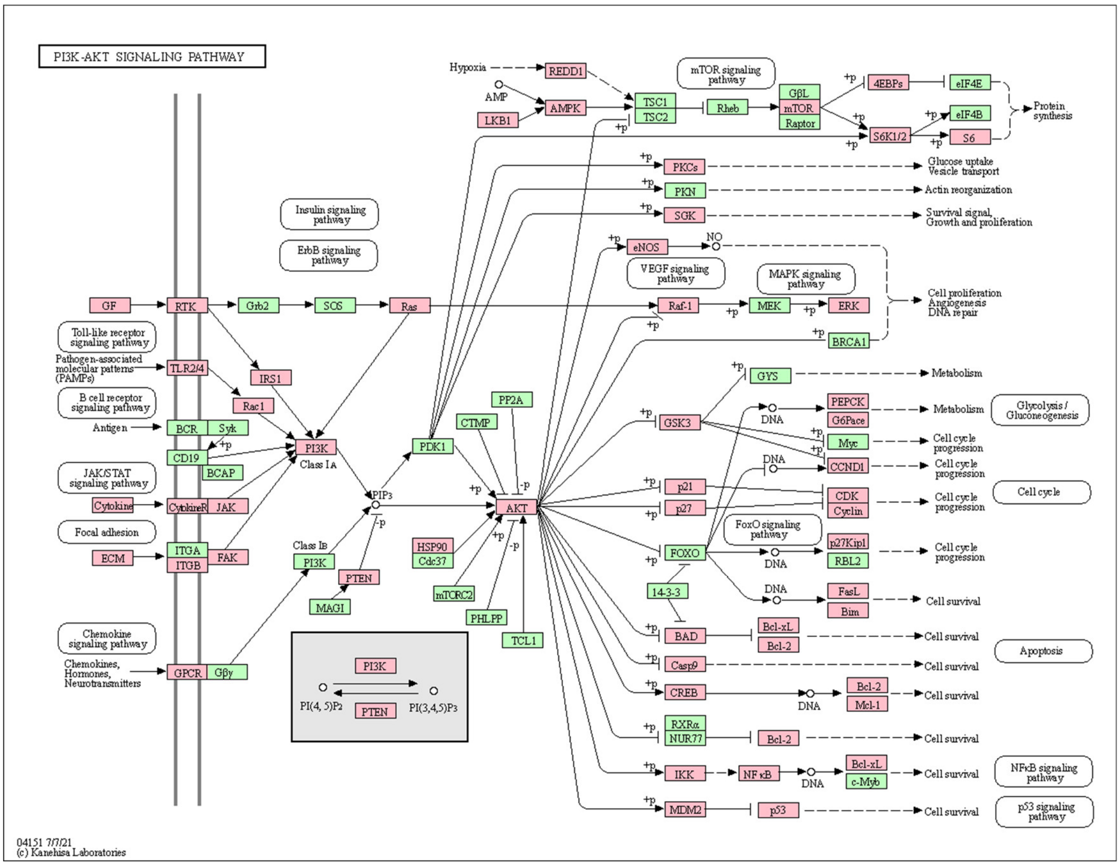

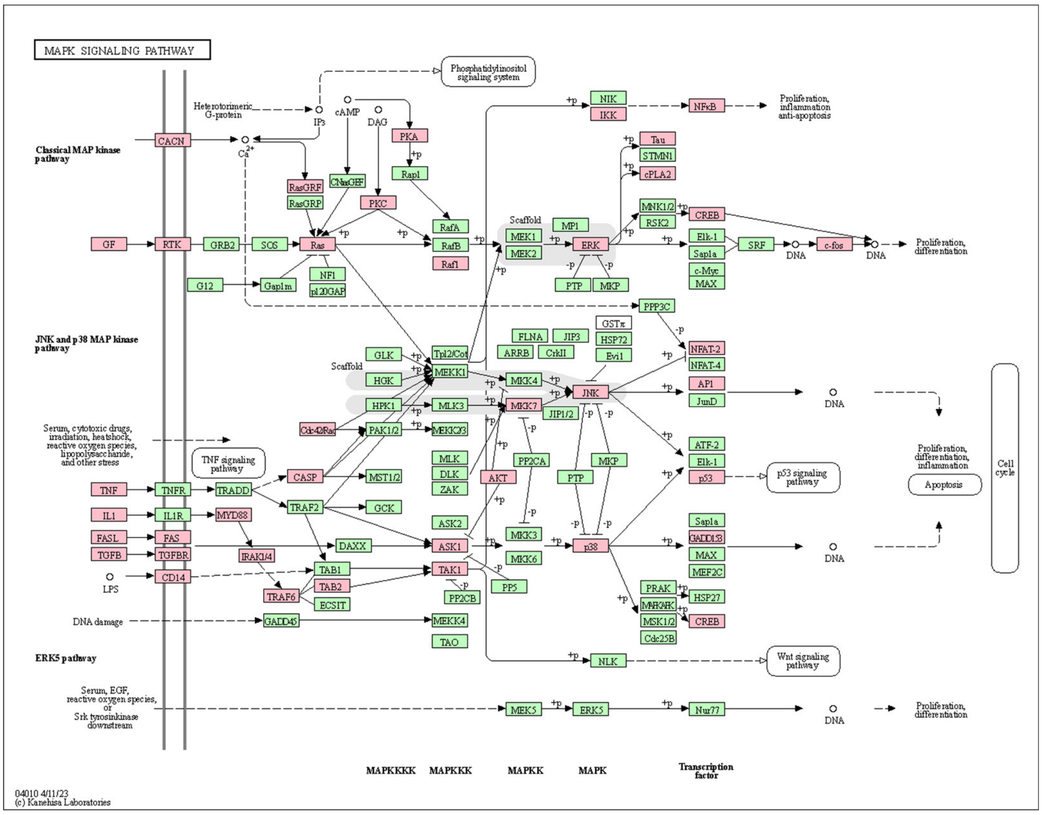

Figure S1. Signaling pathways and targets of CSBHH. The pathway maps were constructed using KEGG mapper. The pink-colored box represents a predicted target gene to interact with CSBHH.

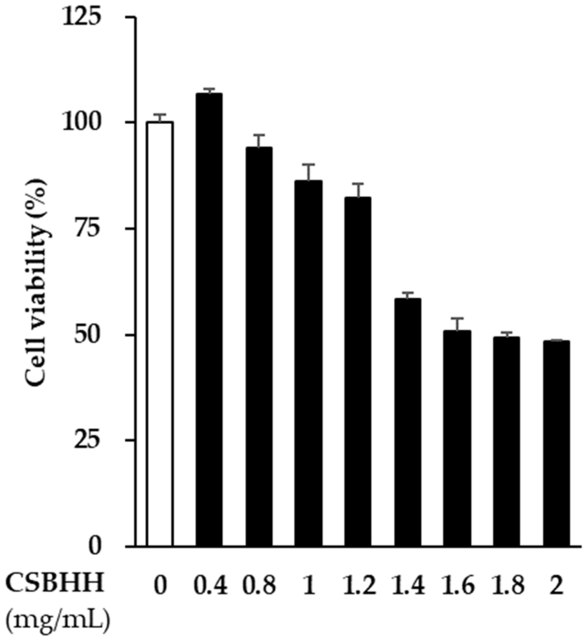

Figure S2. Effect of Chung-Sang-Bo-Ha-Hwan (CSBHH) on NCI-H292 cell viability. The cells (2×10^4 cells/well) were cultured in a 96-well plate for 24 h. Thereafter, the cells were treated with a specific CSBHH concentration for 24 h. Cell viability was determined using an Ez-Cytox kit. Data are presented as the mean ± SEM (n=3).

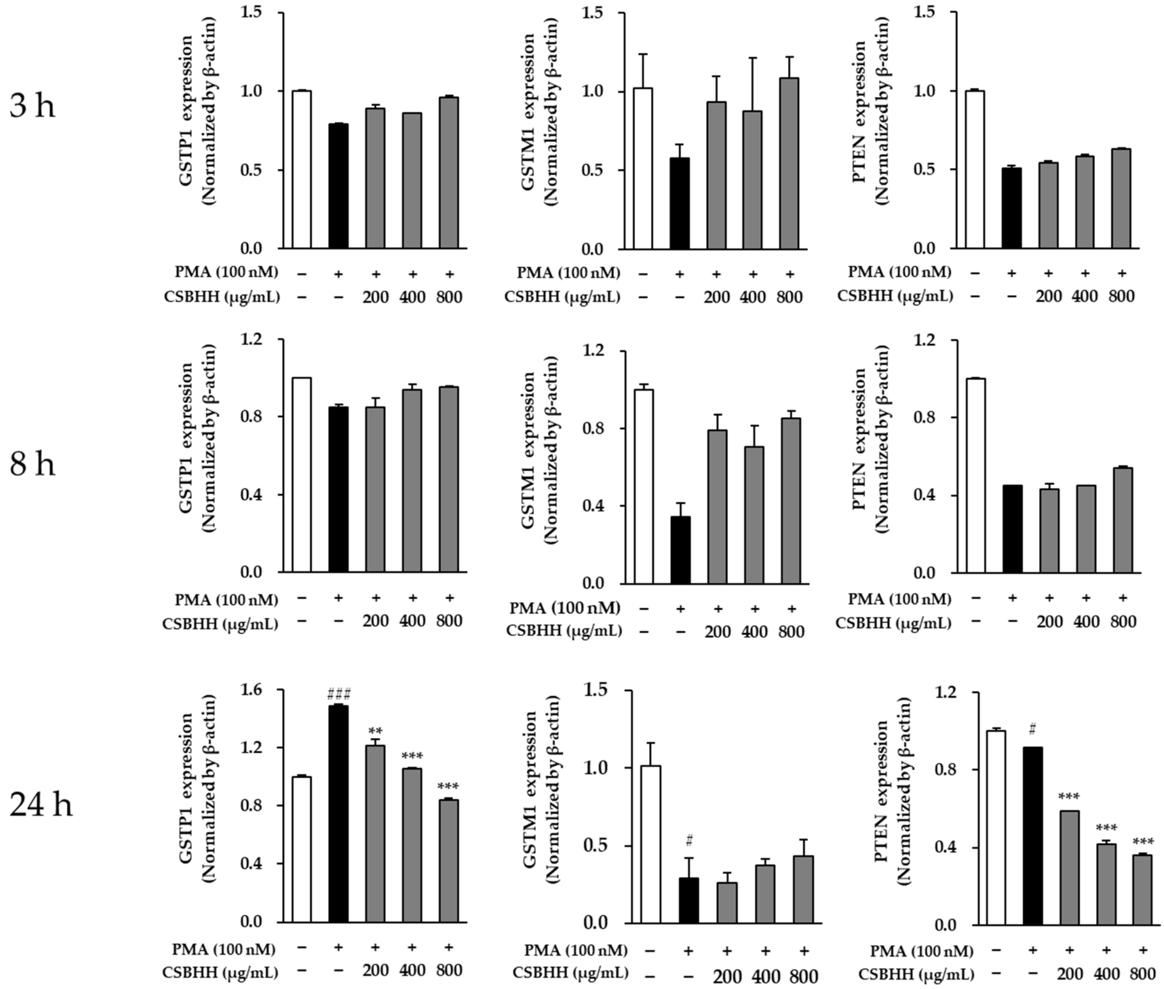

Figure S3. Effect of Chung-Sang-Bo-Ha-Hwan (CSBHH) on phorbol 12-myristate 13-acetate (PMA)-induced GSTP1, GSTM1, and PTEN mRNA expression in NCI-H292 cells. mRNA expression was assessed by qRT-PCR analysis. Data are presented as the mean ± SEM (n = 2). $^{\#}$ $p<0.05$ and $^{\#\#\#}p<0.001$ compared with the untreated cells; $^{**}p<0.01$ and $^{***}p<0.001$ compared with the PMA-treated cells.
